# Concurrent Supra-Postural Auditory–Hand Coordination Task Affects Postural Control: Using Sonification to Explore Environmental Unpredictability in Factors Affecting Fall Risk

**DOI:** 10.3390/s24061994

**Published:** 2024-03-21

**Authors:** Dobromir Dotov, Ariel Motsenyat, Laurel J. Trainor

**Affiliations:** 1 Department of Biomechanics, University of Nebraska Omaha, Omaha, NE 68182, USA; 2 Psychology, Neuroscience and Behaviour, McMaster University, Hamilton, ON L8S 4K1, Canada; ljt@mcmaster.ca; 3 Integrated Biomedical Engineering and Health Science, McMaster University, Hamilton, ON L8S 4K1, Canada; motsenya@mcmaster.ca; 4 Rotman Research Institute, Toronto, ON M6A 2E1, Canada

**Keywords:** attention, balance, coordination, dual task, sonification, supra-postural

## Abstract

Clinical screening tests for balance and mobility often fall short of predicting fall risk. Cognitive distractors and unpredictable external stimuli, common in busy natural environments, contribute to this risk, especially in older adults. Less is known about the effects of upper sensory–motor coordination, such as coordinating one’s hand with an external stimulus. We combined movement sonification and affordable inertial motion sensors to develop a task for the precise measurement and manipulation of full-body interaction with stimuli in the environment. In a double-task design, we studied how a supra-postural activity affected quiet stance. The supra-postural task consisted of rhythmic synchronization with a repetitive auditory stimulus. The stimulus was attentionally demanding because it was being modulated continuously. The participant’s hand movement was sonified in real time, and their goal was to synchronize their hand movement with the stimulus. In the unpredictable condition, the tempo changed at random points in the trial. A separate sensor recorded postural fluctuations. Young healthy adults were compared to older adult (OA) participants without known risk of falling. The results supported the hypothesis that supra-postural coordination would entrain postural control. The effect was stronger in OAs, supporting the idea that diminished reserve capacities reduce the ability to isolate postural control from sensory–motor and cognitive activity.

## 1. Introduction

Falls occur at an alarming rate in the aging population [1]. A quarter of fallers who are older adults experience an injury [2]. According to the CDC, falls are a major cause of injury and a public health issue, with over 800,000 yearly hospitalizations in the US [3]. Mobility is an important component of quality of living in community-dwelling older adults. Mobility is associated with balance in the sense that reduction in the range of environmental contexts in which balance can be safely maintained leads to reduced mobility [4]. This association is present both in the general population of older adults [5] and in specific disorders [6]. Improving mobility requires intervention programs that challenge balance under real-world conditions, and not simple constrained laboratory tasks [5].

Understanding the factors that lead to falls is made difficult by the fact that interaction with the environment during activities of daily living has the potential to interfere with postural control at multiple levels. To begin with, mechanical perturbations can destabilize balance. For example, supra-postural tasks involving manipulating objects with the hands has an unavoidable mechanical influence on postural control. Even the slightest, non-load-bearing mechanical coupling with a moving surface using finger touch is enough to re-organize postural control [7,8]. Yet, factors that interfere with balance are not confined to the mechanical domain.

Among the environmental stimuli that may interfere with postural control, the effects of cognitive distractors have been confirmed extensively. Far from being an isolated autonomous module, postural control of standing balance has been described as cognitively penetrable, meaning that it is influenced by the type and amount of concurrent mental activity [9,10]. Postural sway in older adults becomes more variable in the presence of a cognitive double task [11]. In this context, the unpredictability inherent in complex urban environments is particularly salient, with fall risk increasing in outdoor, compared to indoor, conditions [12]. Urban environments, which can contain uneven surfaces and multiple objects on the ground, are associated with falling and fear of falling in older adults. This is also consistent with the fact that poor visual acuity is a risk factor for falls in older adults [13]. In natural environments, fallers’ visual attention, in comparison to non-fallers’, fixates less on the environment and more on moving distractions such as other passing pedestrians [14].

The visible layout of outdoor environments is not the only sensory modality that allows cognitively distracting stimuli to couple and interfere with balance. The role of auditory stimuli is appreciated less often, even though hearing loss is associated with increased center of pressure parameters [15]. In the laboratory, controlling auditory cues in adults with normal hearing leads to increased postural sway [16], and listening to music in the background leads to reductions in center of pressure velocity [17]. Sway can couple with the spatial displacement of auditory stimuli if the task explicitly demands it [18], and moving sounds can be destabilizing, particularly for people with hearing loss [19]. There are also reports of rhythmic entrainment emerging spontaneously in the presence of repetitive stimuli in the background [20]. Conversely, paying attention to verbal and tonal auditory information, when it is not specific to movement, rhythm, or space, may not be strong enough as a stimulus to affect concurrent standing posture [21,22]. In the wild, hearing loss is independently associated with fall risk [23,24], a reduction in gait speed [25], and decreased mobility [26]. Impaired hearing leads to poor awareness of moving objects in the spatial environment, perhaps implicitly increasing cognitive load and shared attention [23] (p. 20).

It is less known whether older adults are more susceptible to a combination of cognitive–auditory–mechanical interference. Here, we address whether postural control is entrained spontaneously by attention-demanding rhythmic auditory–motor synchronization, and compare healthy older adults to young adults. The synchronization task had an easy and a more challenging condition where the auditory stimuli changed tempo unpredictably. We investigated whether low-cost portable sensors were sufficient to detect differences in task performance and postural fluctuations, thus widening the potential for practical application of postural tasks for testing and training balance control in older adults.

## 2. Materials and Methods

### 2.1. Participants

Eleven students (N = 11, 8/3 female/male) were recruited in the young adult (YA) group (mean age 23.6, range 20–37) and compensated with a coffee gift card. Eleven members of the community (N = 11, 8/3 female/male) were recruited in the older adult (OA) group (mean age 68.7, range 60–73). The mobility and balance abilities of participants were not evaluated extensively, aside from establishing a minimal required level implicit in the inclusion criteria and a questionnaire. Specifically, only participants who did not report any hearing or mobility issues on initial screening questionnaires were invited to participate. An additional criterion for inclusion was the declared ability to stand and walk for fifteen minutes.

### 2.2. Apparatus

**Figure 1:**
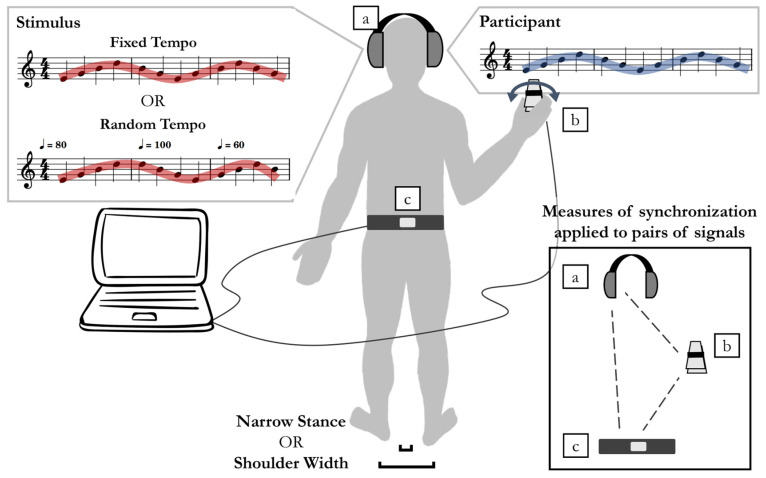
Auditory–motor synchronization task with quiet standing. The main objective for the participants was to control the continuous tone of their instrument to match the pitch and continuous oscillation of a target tone. (**a**) The participant’s tone was played through the channel ipsilateral to the dominant hand; the stimulus, through the contralateral channel. (**b**) Participants controlled the pitch of their instrument by holding a sensor device horizontally and rotating it about an axis roughly aligned with the anterior–posterior axis (in a motion similar to turning a key). (**c**) An additional sensor attached to a soft elastic belt was held flush against the lower back. A Python program running on a laptop computer sampled the sensors, synthesized the left channel tone (stimulus) and the right channel tone (participant-controlled) in real time, and stored all sensor and stimulus values. The stimulus had two levels of predictability: fixed tempo and random tempo. Stance had two conditions of stability: shoulder width and narrow stance. Performance variables measured the rhythmic synchronization and pitch matching between the target tone and the participant’s tone. Inset: Synchronization between the stimulus (**a**) and hand rotations (**b**), and postural fluctuations (**c**) was measured with the cross-wavelet coherence.

A custom-built hand-held device functioned as a sonified tiltmeter: declination from the vertical controlled the pitch of a continuous tone played in the ipsilateral channel of headphones worn by the participant; see Figure 1. Tilt in the range from −90 (left) to +90 (right) degrees was mapped linearly to the tone fundamental frequency in the range from 261.63 to 349.23 Hz. The device used an encased Arduino UNO board (Arduino LLC, Turin, Italy) and an embedded MPU6050 inertial measurement unit (IMU) with a 3D accelerometer and 3D gyroscope (InvenSense, San Jose, CA, USA). This sensor has an on-board digital motion processor for angle and acceleration in real-world coordinates. To sample the postural fluctuations of the participant, a second device identical to the hand-held one (but which did not control a sound) was held in place on the lower back with a soft elastic belt around the torso.

To record the sensor output signals, USB cables connected them to a laptop computer running a real-time GNU/Linux kernel. Custom Python code encoded the sensor tilt signals at a rate between 90 and 100 Hz and used this tilt information signal from the hand-held device to synthesize the pitch of the continuous tone at a 48 kHz sampling rate; see Appendix A. The computer also generated a stimulus tone. The task of the participant was to move the hand-held device so that its sound matched that of the stimulus. Both the stimulus and participant-generated tones were played through over-the-ear open-cup headphones. Participants self-selected a comfortable sound level during practice trials.

The participants stood in front of a desk and held the controller in the air in front and/or to the side of them. The suggested arm and hand posture was similar to holding a key to unlock a door. Participants were asked to find their most comfortable position during practice trials. Participants also self-selected a comfortable grasp of the hand-held controller, either with the thumb and index fingers on its sides or holding it from above and below.

We used hearing and mobility questionnaires to decide whether participants would be invited to participate in the study. PROMIS-Mobility [27] asks participants to select the level of difficulty they experience while performing activities of daily living such as walking, standing up from a chair, and climbing a flight of stairs. A score of 5 (“Without any difficulty”) on all balance-related items was required for participation. The Hearing Handicap Inventory for the Elderly (HHIE-S) asks if respondents fail to understand auditory information in contexts such as talking to family or listening to speech on the radio and in a restaurant [28]. A score of 14 or lower (“Mild-moderate handicap”) was required.

### 2.3. Task

Participants performed two tasks: a simple postural task and a supra-postural task. The simple task was to maintain a quiet standing posture for one minute in either narrow or shoulder-width stance. The supra-postural task involved performing an auditory–motor synchronization task while standing. Participants held and moved the device in their dominant hand to control the pitch of the continuous sound it produced. The goal was to match the pitch of this sound to the pitch of a target (stimulus) tone (see Figure 1). Both tones were played continuously throughout the trial and were pitch-modulated continuously. The participant’s sound was played in the channel ipsilateral to the hand holding the controller. The target was played in the contralateral channel. Its pitch was modulated in a sinusoidal-like manner in the range between 269 and 339 Hz with a fixed or with an unpredictably changing tempo (see Figure 1). More specifically, the modulation was a symmetric triangle wave. Participants controlled the pitch of the tone produced by their device by changing the declination from the vertical of their sensor, with rotation to their left producing lower pitch and rotation to their right producing higher pitch. As the two sounds had identical timbres consisting of a pure tone and two harmonics, ideal synchronization and pitch-matching led to unison between the two channels. The research objectives of our approach were to engage the auditory sensory modality, to be ambulatory and low-cost, to simultaneously challenge both balance and a supra-postural performance, and to allow a comparison of performance between predictable and unpredictable conditions, as the latter is more relevant to fall risk in naturalistic settings.

### 2.4. Design

There were two groups of participants: young adults (YAs) and older adults (OAs). Stimulus predictability was a within-subject factor with two levels: fixed and random tempo. Stance stability was also a within-subject factor with two levels: shoulder width and narrow stance, defined as feet being together.

### 2.5. Stimuli

The auditory stimulus was a continuous tone with two harmonics. Its fundamental frequency changed from 269 Hz (between C4 and C#4) to 339 Hz (between E4 and F4) following a triangle wave. In the fixed-tempo condition, the period of this oscillation was 909 ms. In the random-tempo condition, the modulation period was taken from a uniform distribution with the range of (1,4) s. This period changed in uniform random intervals in the range of (1,5) s.

### 2.6. Procedure

After the participants were seated in a chair in front of an office desk, they heard an explanation of the task, signed an informed consent form, and completed questionnaires on demographic information, history with musical practice and sports, and hearing and motor abilities. All procedures were approved by the ethics board (MREB: #1975). To help their intuition, the task was compared to playing second violin in an orchestra where the first violin was playing in an erratic tempo. A pre-recorded video was played where each condition of the task was demonstrated. Participants performed twenty-two trials, each one minute long. The first two trials were without the target tone to allow familiarization with the novel musical instrument. The following four trials were considered practice trials. After each practice trial, the experimenter explained a figure on the screen which showed the recorded target and the participant’s trajectories. In the remaining 16 trials, each of the four combinations of the stance and stimulus conditions were repeated four times. Trials were blocked by the stimulus condition, and the stance condition was randomized within these blocks. The main part of the experiment lasted approximately 40 min.

### 2.7. Measures

#### 2.7.1. Performance

The level of task performance in each trial was quantified by applying bi-variate methods to the two time series containing the MIDI pitch information from the stimulus and musical instrument device channels. We used two measures: the windowed cross-correlation (*C*) and the root-mean-squared error (*E*) between the two time series. The former is sensitive to rhythmic synchronization with the target; the latter is sensitive to matching not only the temporal pattern but also the exact pitch. The first ten seconds of each trial were excluded from analysis.

Cross-correlation (*C*) is the correlation between two processes after they are time-shifted relative to each other by a range of lags from −τ to τ. The time lag allows *C* to be sensitive to phase synchronization. The windowed approach computes the measure successively over subsections of the trial using a running window. In each separate window, we took the absolute maximum cross-correlation across lags and then averaged across windows to obtain a single value for the entire trial. This approach leaves out the information about the phase of synchronization which was not considered essential for the present purposes. We used non-overlapping windows of length 5 s. The maximum τ which determines the range of lags was set to 1000 ms which was at the time scale of one stimulus cycle. Each time series was z-score-normalized (zero mean and unit variance).

Root-mean-squared error (*E*) is the average magnitude of the difference between signals across time points.

#### 2.7.2. Synchronization with Postural Fluctuations

Lower-back Acceleration Magnitude (A). Postural control is typically studied by way of kinematic or ground reaction force data. Here, the postural data contained lower-back accelerations which required pre-processing. We reduced the 3D accelerations to a single time-series by taking the acceleration magnitude across dimensions, $A_{i}={{(a}_{X,i}^{2}+a_{Y,i}^{2}{+a}_{Z,i}^{2})}^{0.5}$, where *i* was the time sample in the trial [29,30].

**Figure 2:**
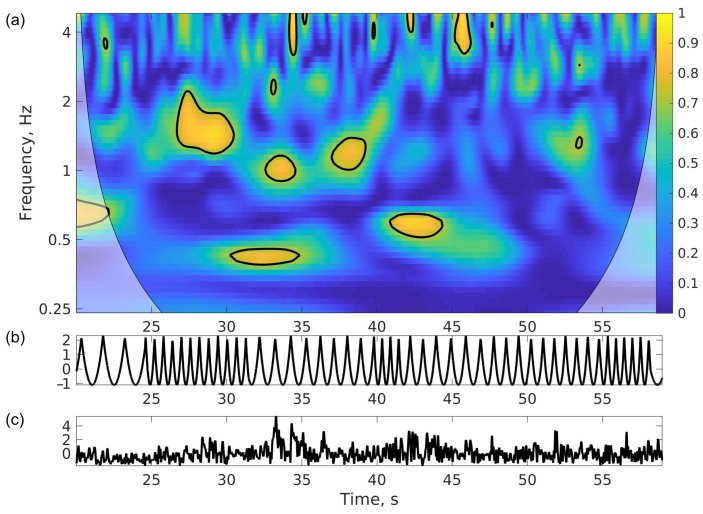
(**a**) Coherence of the cross-wavelet transform of the two time series. (**b**) Squared and normalized stimulus data. (**c**) Normalized postural data (total acceleration at the lower back). Coherence is high for parts of the trial with synchronized movement, coded as a brightness gradient (yellow online). Values exceeding the significance threshold are contoured. The proportion of the area with significant coherence was used as a measure of degree of synchronization in each trial.

Cross-Wavelet Coherence. To measure coupling among postural and supra-postural effectors, we applied a cross-wavelet synchronization method to the stimulus/lower body postural sway and hand/lower body postural sway pairs of sensors. This is an extension of the wavelet transform which is a time–frequency representation of an individual signal; see Appendix B. We used the cross-wavelet transform Matlab toolbox [31]. We applied this method because it has both time and frequency resolution, unlike the cross-correlation, and this makes it sensitive to shifting patterns of coordination present in postural fluctuations which tend to unfold on a range of the frequency spectrum [32]. It estimates two main quantities: common power between two time-series (that is, how much the oscillations in the one match in frequency the oscillations in the other) and coherence (that is, how much they are phase-synchronized); see Figure 2. Here, we concentrated on coherence. It is time- and frequency-resolved because it analyzes signals with localization in frequency bands, similar to spectral analysis, and with localization in time. Importantly, the toolbox implements null-hypothesis testing per time–frequency bin to reduce the chance of reporting meaningless values of spurious synchronization. The cross-wavelet synchronization indicates the areas in the time–frequency space with significant phase-synchronization; see Figure 2. To reduce these rich data to a single value per trial, we used the proportion of the time–frequency space where coherence was significant.

We used the same analysis parameters for each trial and sensor pair. The time series were *z*-score-normalized. We used the Morlet wavelet and limited its scale to the frequency range from 0.25 to 4 Hz. The number of iterations for computing the surrogate null-hypothesis distribution was set to the default of 300. The magnitude of postural accelerations (A) was band-pass-filtered between 0.5 and 6 Hz. Taking the magnitude of the lower-back acceleration flips negative accelerations in the positive domain. For this reason, the stimulus and hand data were full-wave-rectified and squared when analyzing their coupling with acceleration magnitude, meaning that neutral position was zero in the rectified timeseries, and movements both to the left and to the right were positive.

### 2.8. Statistical Analysis

Outcomes from the hearing and mobility questionnaire were compared between groups with a non-parametric Mann–Whitney test. Levels of performance and coupling measures were compared between groups and conditions using ANOVAs. Linear mixed-effects models tested for associations between postural synchronization and performance.

## 3. Results

### 3.1. Hearing and Mobility Questionnaires

Comparing the hearing handicap scores in YAs (*M* = 3.46, *SD* = 5.07) and OAs (*M* = 2.00, *SD* = 4.47) did not show a significant difference (*W* = 80, *n*_1_ = *n*_2_ = 11, *p* = 0.15). When comparing the mobility scores in YAs (*M* = 74.90, *SD* = 0.30) and OAs (*M* = 71.36, *SD* = 5.22), we found that the YA group had significantly higher mobility than OAs (*W* = 85, *n*_1_ = *n*_2_ = 11, *p* < 0.05).

### 3.2. Performance on Matching Hand Movements to the Sound Stimulus

**Figure 3:**
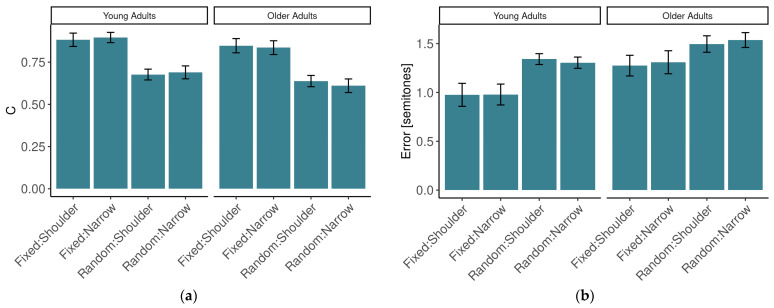
Performance in the main auditory–motor task and its dependence on group and condition. (**a**). Rhythmic synchronization. (**b**) Pitch error.

Pitch-matching error exhibited differences between stimulus conditions and groups; see Figure 3b. Pitch-matching error was worse with the random-tempo stimulus (*M* = 1.42, *SE* = 0.048) than with the fixed-tempo stimulus (*M* = 1.13, *SE* = 0.078), and this was a significant effect [*F*(1,20) = 31.57, *p* < 0.001, η^2^*_G_* = 0.189]. The error was also higher in the OA group (*M* = 1.40, *SE* = 0.085) than in the YA group (*M* = 1.15, *SE* = 0.085), a significant effect [*F*(1,20) = 4.51, *p* < 0.05, η^2^*_G_* = 0.156]. There was no interaction between age group and stance [*F*(1,20) = 3.32, *p* = 0.083]. All other main effects and interactions (stance, age × predictability, predictability × stance, and age × predictability × stance) were not significant [all *F* < 1].

As expected, the measure of rhythmic synchronization *C* in the fixed-tempo condition (*M* = 0.865, *SE* = 0.027) was higher than in the random-tempo condition (*M* = 0.654, *SE* = 0.025) and this was a significant effect [*F*(1,20) = 128.63, *p* < 0.001, η^2^*_G_* = 0.445], see Figure 3a. There was a significant interaction between age group and stance [*F*(1,20) = 4.90, *p* = 0.039, η^2^*_G_* = 0.005]. Specifically, *C* tended to be lower in the shoulder-width condition than in the narrow-stance condition in the YA group [*M*(*SE*) *=* 0.780(0.035) vs. *M(SE)* = 0.793(0.034)] but the relationship was reversed for OAs, where *C* was higher in shoulder-width than in narrow-stance [*M*(*SE*) *=* 0.743(0.035) vs. *M*(*SE*) *=* 0.723(0.034)], although neither of these two comparisons were significant [*p* = 0.224 and *p* = 0.075]. The other main effects and interactions were not significant [all *F* < 1].

### 3.3. Postural Synchronization with the Auditory Hand Movement Task

**Figure 4:**
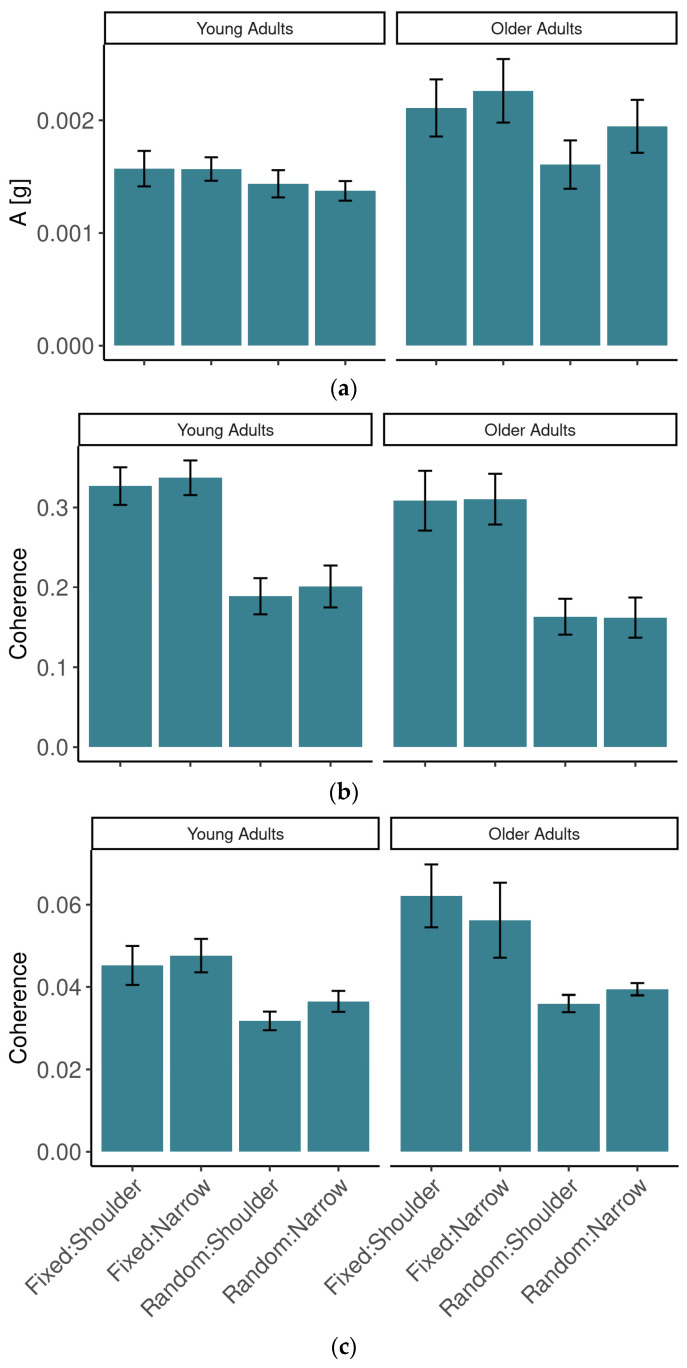
Measures of postural control and synchronization with the main task. (**a**) Lower back acceleration magnitude. (**b**) Cross-wavelet coherence between the stimulus and lower back. (**c**) Cross-wavelet coherence between the hand and lower back.

We were interested in how postural control changed in the context of different conditions of the main task. First, we considered the magnitude of lower-back accelerations (see Figure 4a). *A* was lower in the YA group (*M* = 149 × 10^−5^, *SE* = 18 × 10^−5^) than in the OA group (*M* = 198 × 10^−5^, *SE* = 18 × 10^−5^), but the effect was not significant [*F*(1,20) = 3.78, *p* = 0.066, η^2^*_G_* = 0.139]. There was a significant interaction between age group and stance [*F*(1,20) = 5.20, *p* < 0.05, η^2^*_G_* = 0.013]. Specifically, in the narrow-stance condition in OAs (*M* = 210 × 10^−5^, *SE* = 18 × 10^−5^), *A* was higher than in the shoulder-width condition (*M* = 186 × 10^−5^, *SE* = 19 × 10^−5^), a significant effect (*p* < 0.05), but this comparison in the YA group [*M*(*SE*) *=* 147 × 10^−5^(18 × 10^−5^) vs. *M(SE)* = 150 × 10^−5^(19 × 10^−5^)] did not exhibit a significant difference (*p* = 0.71). Furthermore, *A* in the OA group was higher than in the YA group in the narrow-stance condition (*p* < 0.05) and not different in the shoulder-width comparison (*p* = 0.19). Finally, the main effect of stimulus predictability was significant [*F*(1,20) = 14.29, *p* < 0.01, η^2^*_G_* = 0.051], with *A* being higher with the fixed-tempo stimulus (*M* = 188 × 10^−5^, *SE* = 15 × 10^−5^) than with the random-tempo stimulus (*M* = 159 × 10^−5^, *SE* = 12 × 10^−5^).

The amount of cross-wavelet coherence between postural fluctuations and the target sound stimulus is summarized in Figure 4b. It appeared to be higher in OAs (*M* = 0.049, *SE* = 0.003) than in YAs (*M* = 0.040, *SE* = 0.003), but the difference was not significant despite the presence of a trend [*F*(1,20) = 3.36, *p* = 0.082, η^2^*_G_* = 0.063]. There was a significant effect of stimulus predictability [*F*(1,20) = 12.62, *p* < 0.01, η^2^*_G_* = 0.222], as there was more coherence with the fixed-tempo stimulus (*M* = 0.053, *SE* = 0.005) than with the random-tempo stimulus (*M* = 0.036, *SE* = 0.001). The other main effects and interactions were not significant [all *F* < 1].

The amount of cross-wavelet coherence between postural fluctuations and the hand rotations followed a similar pattern, summarized in Figure 4c. It was higher in OAs (*M* = 0.059, *SE* = 0.003) than in YAs (*M* = 0.049, *SE* = 0.003), a significant effect [*F*(1,20) = 4.62, *p* < 0.05, η^2^*_G_* = 0.088]. There was a main effect of stimulus predictability [*F*(1,20) = 15.36, *p* < 0.001, η^2^*_G_* = 0.176], with more coherence in the fixed-tempo condition (*M* = 0.061, *SE* = 0.004) than the random-tempo condition (*M* = 0.047, *SE* = 0.002). There was also an interaction between stimulus predictability and stance [*F*(1,20) = 8.16, *p* < 0.05, η^2^*_G_* = 0.035]. In the shoulder-width stance, the coherence was larger in the fixed-tempo condition (*M* = 0.063, *SE* = 0.004) than the random-tempo condition (*M* = 0.043, *SE* = 0.002, *p* < 0.001). In contrast, in the narrow stance, coherence in fixed-tempo stimulus trials (*M* = 0.060, *SE* = 0.005) was not different from random-tempo trials even though there was a trend (*M* = 0.051, *SE* = 0.003, *p* = 0.07). The other main effects and interactions were not significant (all *F* < 1).

### 3.4. Association between Postural Synchronization and Auditory Hand-Matching Performance

**Figure 5:**
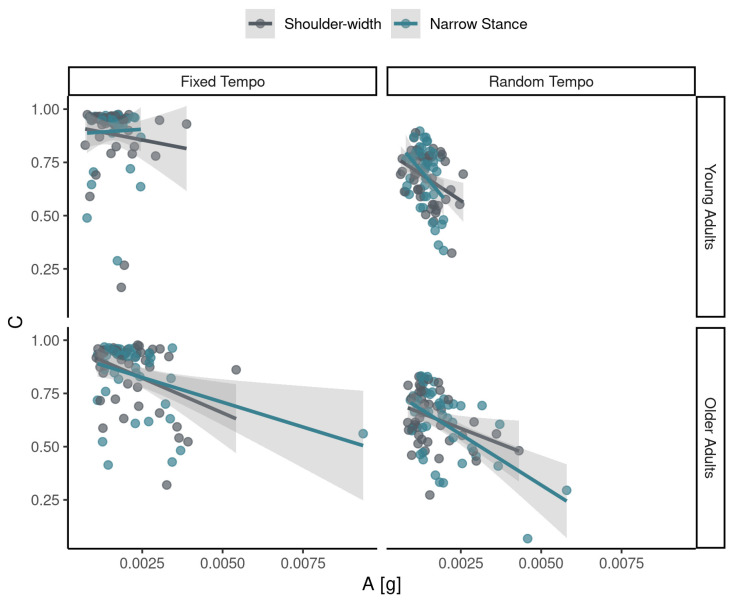
Association between magnitude of lower back accelerations and cross-correlation, the measure of rhythmic synchronization in the main auditory–motor task. The solid lines are linear fits ±95% confidence intervals.

There was a negative association between rhythmic synchronization (*C*) with the stimulus and the magnitude of postural accelerations (see Figure 5). A linear model showed that the association was not significant in YAs (β = 21.54, *SE* = 23.74, *p* = 0.37) but it was significant in OAs (β = −62.15, *SE* = 25.64, *p* < 0.05); see Appendix C.

## 4. Discussion

The present study reported evidence consistent with spontaneous coupling between postural sway during quiet stance and a supra-postural task. The supra-postural task consisted of auditory–motor synchronization between hand movements and an auditory stimulus. The coupling was observed in younger and older participants. There was weak evidence that the level of performance in the synchronization task was linked to the amount of postural response. Specifically, higher postural sway was associated with lower hand synchronization with the stimulus. Given that a mechanical linkage exists between the swaying hand and the swaying body, it is not inherently surprising to observe some level of entrainment between the two. Rotations of the hands can generate momentum which is transferred easily to the upper body because the human body, with a narrow base of support, is mechanically unstable when maintaining a standing balance.

We found that the spontaneous coupling between posture and the supra-postural task was stronger in older adults. This suggests that this group may be more vulnerable to falling if a distracting upper-body task spontaneously entrains postural control to the extent of inducing destabilizing oscillations. Although the present study did not recruit a high-risk population and did not induce falls, the results suggest that the effect of a supra-postural auditory–movement task on postural sway is likely greater in a higher-risk elderly population.

The overall decrease in physical health and increased multidimensional risk of adverse outcomes in older adults has been described as frailty. Frailty is associated with a risk of falling in circumstances of divided attention [33,34]. To explain why testing of older adults sometimes does not reveal a potential deficit until the participants are exposed to a challenging task and divided attention, the cognitive reserve hypothesis refers to the relative availability of (neural) resources to accommodate habitual and unexpected peak activity [35,36,37].

The reserve hypothesis was formulated primarily to address cognitive phenomena, yet its overall relevance to motor control and sensory–motor performance is striking. In movement science, the redundancy of degrees of freedom is a fundamental problem of theoretical motor control. For example, it is possible to execute the same kinematic trajectory of the hand-held sensor by swaying either the hand, the arm, the whole upper body, or any combination thereof. This redundancy can be seen as a computationally challenging control problem because the number of variables to be controlled is much higher than the number of dimensions of the task that participants are performing. Yet, when reformulated as a principle of abundance, this can be seen as a possibility for adaptive response to unexpected perturbations [38]. From this perspective, the separate levels of control associated with manual rotations and balance should benefit from orthogonalizing variability associated with the two tasks to spare balance from perturbations [39]. This separation may be more difficult in older adults, as suggested by the present result. Their strategy to perform the challenging task may have been to allow posture to sway along with the hand, even though the task was for standing posture to be still, not free. This response was subtle and intermittent in the present study, see Figure 2, and did not destabilize participants. It is possible, however, that in a busy natural environment, even a subtle response could be maladaptive by diminishing the reserve capacities necessary to deal with other unexpected balance perturbations.

The interaction between divided attention and postural control is likely to be mediated not only by motor but also by perceptual constraints. Cognitive activity can increase the amount of postural sway, and evidence indicates that it can be related either to general attentional interference or specific interference with processes for spatial awareness [10]. Consistent with the latter, when supra-postural activity involves sensory–motor coordination, as in the present study, postural sway may be recruited to support stimulus-focused sensing [40].

We presented multiple reasons for evaluating balance in situations involving cross-modal, multi-tasking, and unpredictable elements to detect elevated risk of falling. This is particularly important for frail populations in which a loss of underlying capacity is suspected. We showed how this can be measured using a portable and affordable apparatus, and a self-explanatory task involving synchronizing hand movements to an auditory stimulus, that can be deployed in ambulatory settings. The generality of this approach is yet to be evaluated, however, because we only employed a constrained postural task, namely maintaining standing balance in a static environment. The power of auditory stimuli from the environment to impact balance and mobility in ecological settings is an under-explored field of research with important implications. Furthermore, there is little mechanistic understanding of why and how auditory sensory information reaches postural control. There is increasing evidence from humans and other vertebrates, however, that the auditory cortex is inherently integrated into feedback loops with motor areas of the brain, specifically by receiving action-relevant motor signals to help it filter out self-produced sounds [41].

The association between sound and posture has the potential to be exploited to design innovative training, intervention, and mobility aid strategies. Several applications use sonification of biomechanical variables, whereby real-time biofeedback as to, for instance, the angle of a joint, is given through sound. Examples include sonified feedback for postural stabilization during challenged standing [42], knee-angle feedback during walking [43], gait improvement in Parkinson’s disease [44], and sensory substitution for visual impairment [45,46]. From the present results, we can add that sonifying movements not only holds promise as a form of real-time biofeedback, but also holds promise for investigating how multi-tasking affects the cognitive and motor resources needed for safe real-world mobility.

## Figures and Tables

**Figure A1:**
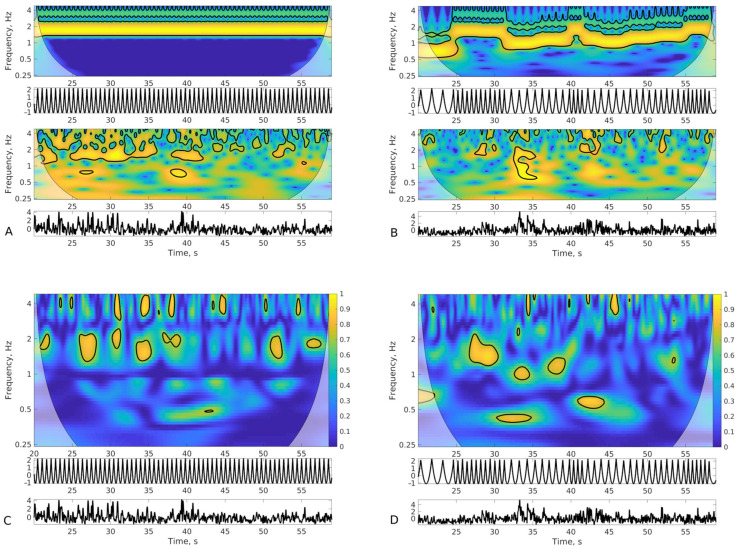
Cross-wavelet analysis of the squared stimulus oscillation data and the lower back acceleration magnitude ((**A**), a representative fixed-tempo trial; (**B**), a representative random-tempo trial). The magnitude of the individual wavelet transform is color-coded along with the time series as an inset underneath it (first row: stimulus; second row: lower back). ((**C**), same trial as in (**A**); (**D**), same trial as in (**B**)). The cross-wavelet coherence is shown. In (**A**–**D**), coherence is brightness-coded (color online) on the same scale and the full color bars are given for reference. The values inside the contoured regions (yellow online) are significant. For a single measure of degree of coupling in each trial, we used the proportion of significant area relative to the full time–frequency space.

## Data Availability

The data that support the findings of this study are available from the corresponding author, D.D., upon reasonable request.

## Appendix A. Sonification Engine

One of the basic challenges in real-time digital sound synthesis is to avoid audible clicks produced by discontinuous shifts in the waveform occurring between packets of the audio buffer. A second problem is to scale the signal amplitude with respect to frequency to achieve equal loudness; inverse scaling of amplitude approximates the equal loudness contour in the range from middle to low pitch [47]. We satisfied both requirements using the so-called canonical–dissipative oscillator [48,49].

(A1) $$\ddot{x}=-\omega^{2}x-\gamma \dot{x}\left(0.5\omega^{2}x^{2}+0.5{\dot{x}}^{2}-\beta \right)$$

This oscillator is suitable for synthesizing pure tones because its state variable is a harmonic wave. Its trajectory settles on a parametrically controlled attractor which determines its frequency and amplitude. This means that the system can achieve smooth oscillations and smooth transitions between parametrically controlled frequencies despite the fact that the control parameter arrives discretely from the motion sensor sampled non-synchronously at a much lower rate. Importantly, the amplitude of the oscillator scales inversely with frequency [50]. This automatically solves the problem of determining the amplitude–pitch relation in real time to achieve reasonable psychophysical properties of the synthesized tone. In complexity, this approach stands between wavetables and nonlinear physical models of musical instruments [51,52]. The dynamical system A1 was Euler-integrated at 48 kHz, the rate of sound synthesis, with parameters β = 1000 and γ = 1 selected for fast convergence to the attractor controlled by the frequency control parameter ω.

The sensor angle $\theta_{x}$ was mapped linearly to the MIDI space of musical notes, $n=\left(\frac{\theta_{x}}{90}+1\right)2.5+60$, and then to frequency in radians, $\omega =2\pi (440\times 2^{\left(n-69\right)/12})$. In short, movement in the range (−90,+90) degrees declination from the vertical was mapped to the MIDI notes range (60,65) corresponding to (C4,F4). Two harmonics were added by running parallel oscillators at 2ω and 3ω.

## Appendix C. Linear Model Table

**Table A1:** Association between cross-correlation *C* as a measure of auditory–motor rhythmic synchronization and magnitude of lower back accelerations *A* as a measure of postural control. The linear mixed-effects model was fitted incrementally by adding terms up to the maximal model, C_ij_ = β_0_ + σ_0i_ + β_1_A_ij_ + β_2_O_ij_ + β_3_RT_ij_ + β_4_NS_ij_ + β_5_A_ij_O_ij_ + σ_ij_. *i* is individual and *j* trial. *Y* is young adults, *O* older adults. β parameter estimates (standard error) are shown in the upper half of the table. β_0_ corresponds to *C* of young adults with fixed-tempo stimulus and shoulder-width stance. Subsequent parameters estimate how *C* changes as a function of the predictor A and switching the respective age group and condition. The final selected model is in bold. * *p* < 0.05, † *p* < 0.01, ‡ *p* < 0.001.

	Model 1	Model 2	Model 3	Model 4	Model 5	Model 6
β_0_: Y, Fixed tempo, Shoulder-width	0.760 (0.024) ‡	0.745 (0.034) ‡	0.771 (0.039) ‡	0.943 (0.034) ‡	0.943 (0.035) ‡	**0.862 (0.049) ‡**
β_1_: A		8.325 (13.337)	9.897 (13.418)	−31.351 (9.693) †	−31.358 (9.735) †	**21.544 (23.744)**
β_2_: O			−0.058 (0.048)	−0.038 (0.043)	−0.038 (0.043)	**0.059 (0.060)**
β_3_: Random tempo				−0.221 (0.011) ‡	−0.221 (0.011) ‡	**−0.218 (0.011) ‡**
β_4_: Narrow stance					0.000 (0.011)	**0.002 (0.011)**
β_5_: A in O						**−62.153 (25.639) ***
AIC	−277.907	−276.264	−275.720	−528.183	−526.183	**−529.953**
BIC	−266.316	−260.810	−256.401	−505.001	−499.137	**−499.043**
Log-Likelihood	141.953	142.132	142.860	270.091	270.091	**272.976**
Number of observations	352	352	352	352	352	**352**
Number of random effects groupings	22	22	22	22	22	**22**
Variance of individual intercepts σ_0i_	0.011	0.012	0.011	0.009	0.009	**0.010**
Variance of residual σ_ij_	0.023	0.023	0.023	0.011	0.011	**0.010**
